# Research on Key Technologies for the Static Measurement of Railway Track Smoothness

**DOI:** 10.3390/s23218658

**Published:** 2023-10-24

**Authors:** Dabao Lao, Fang Wang, Yongbin Quan, Yukun Liu

**Affiliations:** 1 College of Automation, University of Science and Technology Beijing, Beijing 100083, China; 15011024094@163.com (F.W.); 18931525608@189.cn (Y.L.); 2 Beijing Engineering Research Centre of Industrial Spectrum Imaging, Beijing 100083, China; 3 Shunde Innovation School, University of Science and Technology Beijing, Foshan 528399, China; 17724223994@163.com

**Keywords:** track smoothness detection, liquid wedge, ring grating, laser spot image processing

## Abstract

In this study, a static railway track smoothness detection system based on laser reference, which can measure various track smoothness parameters by using multiple sensors, is proposed. Furthermore, in order to improve the measurement accuracy and stability of the system, this paper also conducted three key analyses based on the static track measurement system. By using a liquid double-wedge automatic compensation device to compensate the horizontal angle of the beam, a mathematical model of liquid double-wedge automatic compensation was established. Then, by using an optical ring grating system to ring-grate and characterize the laser spot, the collimation efficiency of the system was improved when measuring at long distances. For the special ring grating spot image, an adaptive image processing algorithm was proposed, which can achieve sub-pixel-level positioning accuracy. This study also conducted a field measurement experiment, comparing the experimental data obtained via the static track measurement system with the results of existing track measurement products, and verifying that the static track measurement system has high measurement accuracy and stability.

## 1. Introduction

Railway tracks will produce different degrees of deformation and wear in the long-term operation of trains. This phenomenon is called track degradation [1]. The main reasons for track degradation include mechanical degradation and chemical degradation. Mechanical degradation refers to the fatigue, wear, and plastic deformation of track materials caused by vehicle loads, track structures, et cetera, resulting in a decrease in the strength, stiffness, and stability of the track, and a change in the vehicle-track interaction model. Chemical degradation refers to the oxidation, corrosion, and other reactions between substances, such as water and oxygen in the air, and track materials. Prolonged extreme weather can also lead to a decrease in the durability and corrosion resistance of the track [2,3]. Track degradation can reduce the safety, comfort, efficiency, and reliability of railways; therefore regular detection and maintenance is required [4].

At present, the detection of railway tracks is divided into dynamic detection and static detection. Dynamic detection refers to a method of real-time monitoring and evaluation of the geometric shape and structural performance of the track during high-speed train operation, which can reflect the dynamic response and change patterns of the track. Dynamic detection generally uses large high-speed rail inspection vehicles, which collect track information through sensors such as lasers and ultrasounds, and can cooperate with GPS global positioning system receivers to provide measurement data, including position information. Static detection refers to the use of manual measurement or small track detection equipment to measure the smoothness parameters of the track without providing wheel load, or including the detection of the gauge, longitudinal level, alignment, cross level, and twist [5]. The research conducted in this article is also based on the device that utilizes the collimation of the laser to detect track smoothness. The core detection quantity of this device is alignment and longitudinal level, as this is very important in train operation. Alignment and longitudinal level refer to the projection deviation of the center line of a single track relative to the initial design in the horizontal and vertical directions, respectively. The track alignment deviation will cause the train to generate lateral force beyond the design threshold during operation, which will cause accidents, such as train rollover and derailment, in sections with large bending degree. Longitudinal level deviation will cause the train to generate extra vertical force, reducing the stability and braking effect of the train operation. Therefore, studying the key technology of railway track smoothness detection and improving the detection level of railway track smoothness are important topics to ensure the safety and reliability of railway transportation [6,7].

At present, issues related to static detection of track smoothness mainly rely on the use of advanced detection instruments and methods to accurately evaluate the level of track smoothness and adjust and optimize the geometric state of the track in a timely manner. In recent years, with the development of laser technology and precision measurement instruments such as theodolites, a series of developments have been made in the fields of static detection of track smoothness and optical precision measurement. L.Yao [8] and others use a laser tracker as the main sensor for obtaining the coordinates of left- and right-track points to detect potential track static irregularities, and the feasibility of using a laser tracker for track static detection was demonstrated through comparative experiments. C.Qijin [9] and others integrated an Inertial Navigation System (INS) with a geodetic surveying apparatus, and design a modular TGMT system based on aided INS, which can be configured according to different surveying tasks and could operate in mobile surveying mode to significantly improve the surveying efficiency. Burak Akpinar and Engin Gülal [10] have designed a new railway track geometry measurement system that combines LVDT, inclinometer, GNSS receiver, and total station design, which can be used to replace classical geodetic methods. By using this measurement system, track gauge, superelevation, and track axis coordinates can be quickly determined. In the field of optical research related to lasers, Iwasińska-Kowalska [11] and others proved the feasibility of using a liquid-filled prism with variable angles to stabilize the laser beam for interference displacement measurement and conducted experimental verification with different liquid media, achieving a deflection of 90 urad for the laser beam, with a resolution of 0.1 urad. Chern-Sheng Lin [12] and others proposed a new spot positioning method for low spot positioning accuracy during laser welding, which first uses a convolutional neural network to select the spot area and then uses template matching to accurately reposition the spot center, showing that the positioning accuracy of this method can reach 0.14 um. In addition, the issue of track detection is related to multiple fields. L.Yifan [13,14] proposed a new method consisting of time-frequency ridge extraction and improved multi-step probability method for more accurate Instantaneous Angular Frequency (IAF) estimation. This method integrates a new cost kernel function and an adaptive search region detection principle and fully exploits information within the vibration response while promising robustness to the variations of the running regimes. It is expected to introduce the field of track detection to solve some prominent problems.

This study focuses on the common problems in the field of laser measurement for track static detection and proposes combining three key technologies of liquid wedge automatic compensation, laser ring grating collimation, and spot image digital processing to improve the detection accuracy and stability of the current track smoothness. It also provides a new solution for solving various outstanding problems, such as cumbersome operation process, easiness to be affected by environmental conditions, high cost, et cetera, of the current static track detection equipment.

## 2. Principle of Static Detection of Track Smoothness Based on Laser Datum

**Figure 1:**
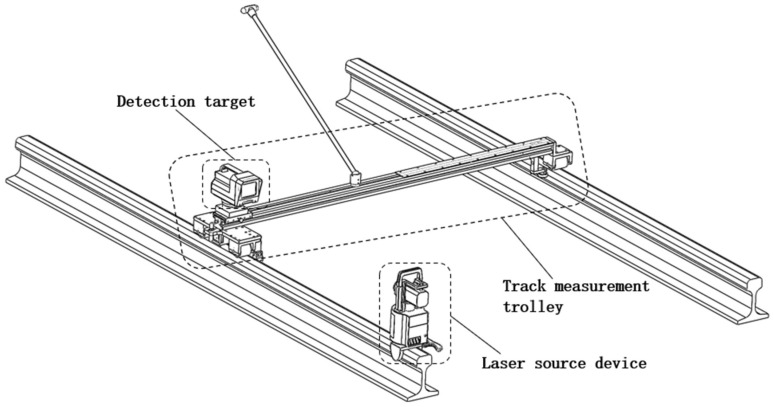
A novel track smoothness detection device.

This study is based on a novel static laser long chord track smoothness detection system, which uses laser reference to measure the alignment and height smoothness of the track and also uses displacement, tilt, and photoelectric switch sensors to assist the measurement. The system structure is shown in Figure 1.

When using this device to perform track smoothness detection, the entire measurement system needs to be collimation: fix the laser source device horizontally at one end of the track to be measured, place the track measurement trolley at the other end, and adjust the horizontal base of the laser source device so that the emitted beam can be vertically projected onto the detection target. At this time, the position of the track measurement trolley is the collimation point of the entire measurement system; the straight line extending horizontally from the collimation point to the laser source direction is defined as the measurement reference line, and the spot position projected on the detection target is defined as the reference coordinate. After collimation, the track measurement trolley is pushed towards the laser source direction, and the spot position projected on the detection target will change according to the current track status. The horizontal offset of the spot position relative to the reference coordinate is the alignment smoothness parameter, and the vertical offset is the longitudinal level smoothness parameter. In actual measurement, it is also necessary to pay attention to the influence of the track elevation difference on the measurement of the alignment and longitudinal level. This track measurement system can measure the track gauge by using a linear displacement sensor and measure the tilt angle by using a tilt sensor to calculate the elevation difference in the track measurement points, compensating the calculation results to the alignment and longitudinal level measurement.

**Figure 2:**
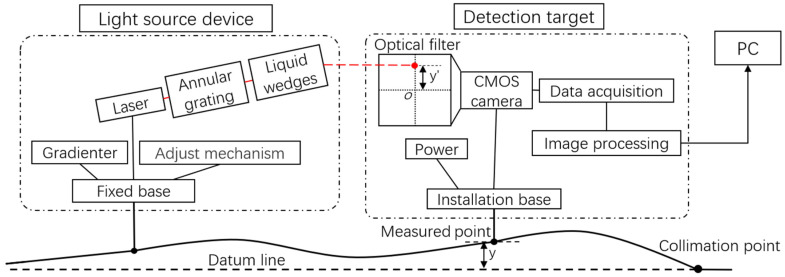
Track longitudinal level detection principle diagram.

The principle of the static track measurement system for measuring the longitudinal level is shown in Figure 2. Assuming that the spot reference coordinate measured at the completion of collimation is $\left(x_{0},\;y_{0}\right)$, the track measurement trolley is pushed to the measured point in Figure 2. At this time, the longitudinal level of the track is represented by *y* in Figure 2, and the vertical offset of the spot center coordinate from the origin o of the camera image is y′. Then, the height smoothness parameter of the track at this measured point can be expressed as:

(1) $$y=y\prime -y_{0}$$

**Figure 3:**
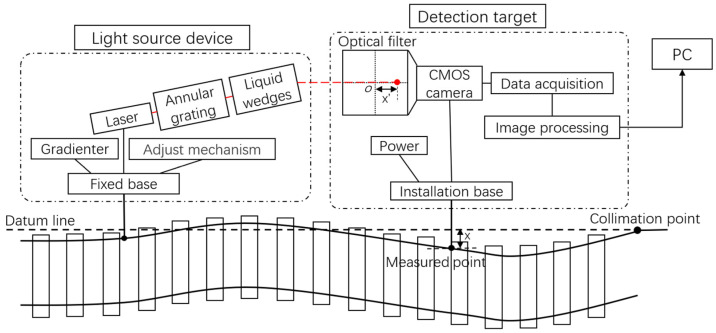
Track alignment detection principle diagram.

The principle of the static track measurement system for measuring the alignment of the track is shown in Figure 3. Similar to the principle of measuring longitudinal level, the alignment of the track at the measured point can be expressed as:

(2) $$x=x\prime -x_{0}$$

## 3. Key Technology of Track Smoothness Detection

### 3.1. Liquid Wedge Automatic Compensation Technology

#### 3.1.1. The Principle of Automatic Compensation for Liquid Wedge

The liquid wedge is a wedge-shaped optical refractive element with liquid as the medium. In order to solve the problem of the horizontal tilt angle of the beam caused by the installation tilt of the laser source device, this paper designed an optical instrument that uses a double-layer liquid wedge to automatically compensate the deflection angle of the light source output beam [15]. The compensator is then installed inside the laser source device. When the attitude angle of the laser source device changes slightly, a deviation angle is generated between the output light path and the measurement reference line. The compensation liquid inside the compensator forms an absolutely horizontal free liquid surface due to the gravity effect [16]. At this time, the compensation liquid can be regarded as a liquid wedge with a very small wedge angle. The laser beam emitted by the laser source will adjust the light path toward the direction of the measurement reference line when passing through this liquid wedge, thus achieving the automatic compensation effect of the whole system light path [17,18].

**Figure 4:**
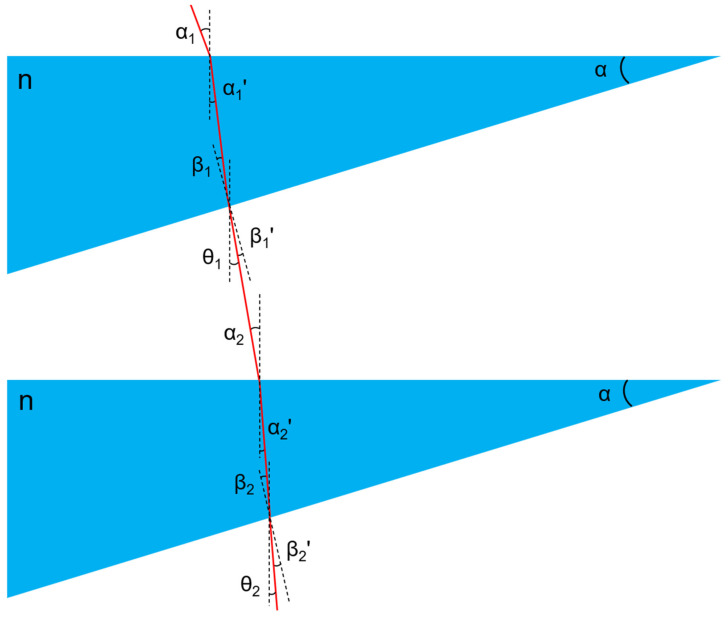
Liquid double optical wedge automatic compensation principle diagram.

The calculation model of the internal optical path of the compensator is shown in Figure 4, and the characteristics and representations of the physical quantities involved are shown in Table 1. Assuming that the angle between the outgoing beam and the measurement reference line is α at this time, the gravitational effect will also cause the compensation solution to produce a wedge angle of alpha size, and the incidence angle of the incident beam entering the upper surface of the liquid light wedge is also α.

According to the principle of liquid level:

(3) $$\alpha_{1}=\alpha$$

(4) $$\theta_{1}=\alpha -\beta_{1}^{\prime }$$

According to the law of refraction and the principle of similar triangles, the following relationship can be obtained:

(5) $$\alpha_{1}=n\alpha_{1}^{\prime }$$

(6) $$n\beta_{1}=\beta_{1}^{\prime }$$

(7) $$\alpha_{1}=\alpha_{1}^{\prime }+\beta_{1}$$

From Equations (3)–(7), we find:

(8) $$\theta_{1}=\frac{\left[1-{\left(n-1\right)}^{2}\right]\beta_{1}}{n-1}=\frac{\left[1-{\left(n-1\right)}^{2}\right]\alpha_{1}}{n}$$

Solving Equation (8) for $\theta_{1}=0$, we obtain:

(9) $$\left\{\;\begin{array}{c} n_{1}=2\\ n_{2}=0 \end{array}\right.$$

The above calculation results show that when the refractive index of the compensation liquid is 2, the complete compensation for the inclination angle of the incident light can be achieved. But as liquids with such a high refractive index basically do not exist, hence, a second layer of compensation liquid is added to perform a secondary compensation for the beam. Similar to the compensation principle of the first layer of compensation liquid, the following relationship can be obtained according to the law of refraction and the principle of similar triangles:

(10) $$\alpha_{2}=\theta_{1}$$

(11) $$\alpha_{2}=n\alpha_{2}^{\prime }$$

(12) $$n\beta_{2}=\beta_{2}^{\prime }$$

(13) $$\alpha =\alpha_{2}^{\prime }+\beta_{2}$$

(14) $$\theta_{2}=\alpha -\beta_{2}^{\prime }$$

The joint solution of Equations (10)–(14) produces:

(15) $$\theta_{2}=\frac{\alpha_{2}}{n}+\left(1-n\right)\beta_{2}$$

It is then obtained by combining Equations (8), (11) and (13):

(16) $$\beta_{2}=\frac{2\left(n-1\right)\alpha_{1}}{n}$$

Then, through the joint solution of Equations (8), (10), (15) and (16), we obtain:

(17) $$\theta_{2}=\frac{1-\left(2n+1\right){\left(n-1\right)}^{2}}{n^{2}}\alpha_{1}$$

Simplified (17) produces:

(18) $$\theta_{2}=\left(3-2n\right)\alpha_{1}$$

It can be seen from Equation (18) that when the refractive index of the compensation solution is 1.5, the inclination angle of the outgoing beam can be fully compensated ($\theta_{2}=0$).

#### 3.1.2. Verification Experiment of Liquid Wedge Automatic Compensation

**Figure 5:**
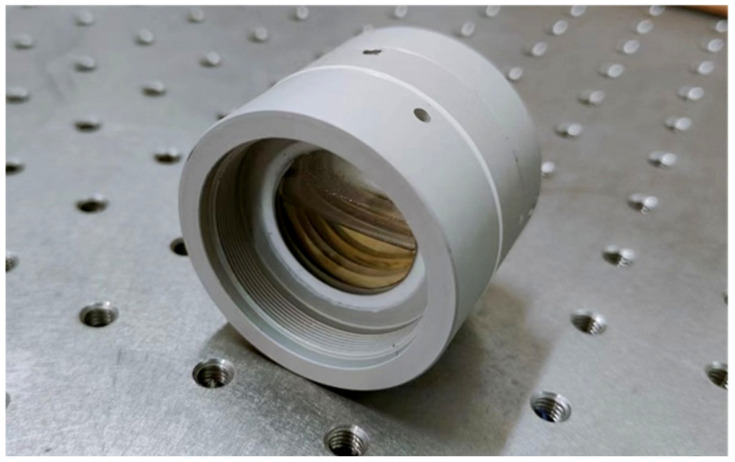
Liquid wedge compensator with secondary compensation liquid.

This paper designs a double-layer liquid wedge compensator based on the above principles, as shown in Figure 5. The compensation liquid inside the compensator is composed of a mixture of water and phthalate, with a refractive index of 1.5, which meets the complete compensation condition for the inclination angle of the beam.

**Figure 6:**
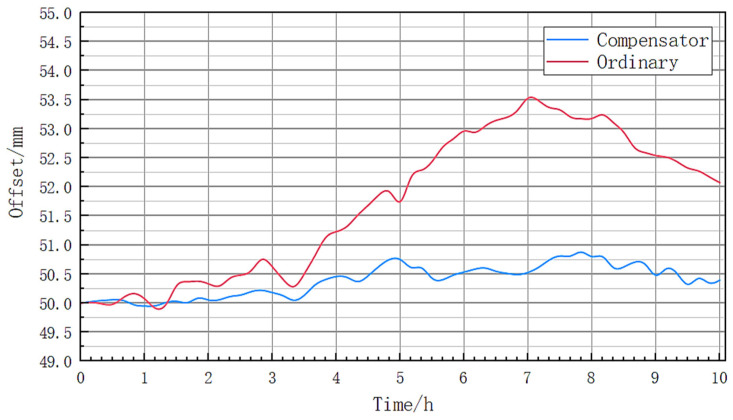
Spot position offset map.

The laser source installed with the compensator was placed on the same platform as the regular laser source, and the spot coordinates of the two laser sources were measured using a vertical optical scale. The scale is 10 m away from the light source, and there is no difference between the two laser sources used in the experiment except for the compensator. The scale values of the initial positions of the two laser spots are the same. The positions of the two laser spots are recorded every five minutes, and the experimental results shown in Figure 6 are obtained.

From Figure 6, it can be seen that the ordinary laser source can maintain a deflection error within 0.25 mm within 1.5 h. As the experiment time increases, the variation of the deviation of the laser spot intensifies, with the maximum deviation appearing at 7 h, reaching 3.601 mm. Throughout the entire experiment process, the range of variation of the deviation of the ordinary light source is 3.74 mm. The laser source equipped with the compensator can maintain the deflection error within 0.25 mm within 3.5 h, with an offset variation range of 0.97 mm. It is clear that, whether in short-term or long-term measurements, laser source devices with compensators have shown significant improvements, especially when the mapping distance is large.

### 3.2. Laser Ring Gate Collimation Technology

In the long-distance measurement system based on the laser reference, in order to ensure the measurement’s accuracy, it is often necessary to cooperate with the optical system to collimate and focus the laser beam. But the collimation efficiency of the optical system is limited, and frequent focusing during measurement will also introduce focusing operation errors. For the orbital measurement system mentioned in this paper, good spot quality is an important prerequisite to ensure measurement accuracy. In order to solve the long-distance measurement time spot when it is too large, has uneven brightness, edge blurring, and other problems introduced by the spatial phase modulator, this optical system will change the phase information of the beam everywhere so that the beam forms a set of light and dark ring spots, known as an optical ring grid. The center bright spot formed after passing through the ring grid still maintains high optical quality, and on this basis, the divergence angle is enlarged to enhance the ring feature to highlight the central bright spot. Then, the position measurement problem of the entire spot is transformed into the measurement of the position of the minimum bright spot in the center of the ring spot [19]. In this orbit measurement system, the use of this technology eliminates the necessary focusing work of the general optical measurement system, removes the accuracy limitation of the focusing error on long-distance optical measurement, and greatly improves the collimation efficiency, measurement accuracy, and anti-interference of the orbit measuring system, thus simplifying the subsequent image processing work for the position measurement of the smallest bright spot in the center [20].

**Figure 7:**
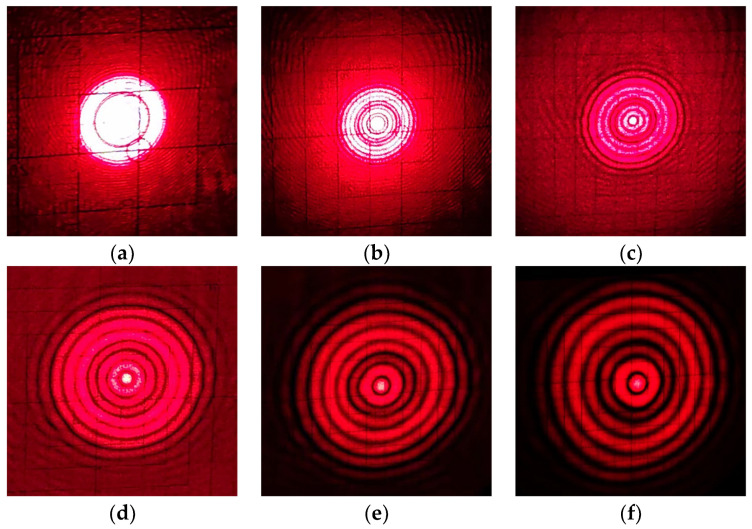
Ring grid spot at different mapping distances: (**a**) 10 m; (**b**) 20 m; (**c**) 50 m; (**d**) 100 m; (**e**) 150 m; (**f**) 200 m.

The laser source equipped with the ring grid system was tested at a long distance, and six images of circular light spots at different mapping distances were collected using an optical calibration plate, as shown in Figure 7a–f.

Based on the experimental data and Figure 7a–f, the following conclusions can be drawn:

(1) When the measurement distance increases, in addition to the geometric increase in the outer diameter of the light spot, the light intensity of the light spot becomes more dispersed and the diffraction effect becomes more significant. For circular light spots, increasing the measurement distance makes their circular characteristics more obvious, and the minimum bright spot at its center position is least affected by energy dispersion effect, which can also maintain a high quality in long-distance measurement.
(2) Based on the measurement results of the optical scale plate, between around 50 m and 100 m, the outer diameter of the smallest bright spot increases by 0.3 mm, and between around 100 m and 200 m; the outer diameter change in the smallest bright spot is stable within 0.1 mm. It can be considered that the ring grid light source system retains strong optical collimation effect even after eliminating the focus operation, which also makes to subsequent spot coordinate extraction operations more convenient.

### 3.3. Spot Image Digital Processing Technology

#### 3.3.1. Digital Processing Process of Light Spot Images

**Figure 8:**
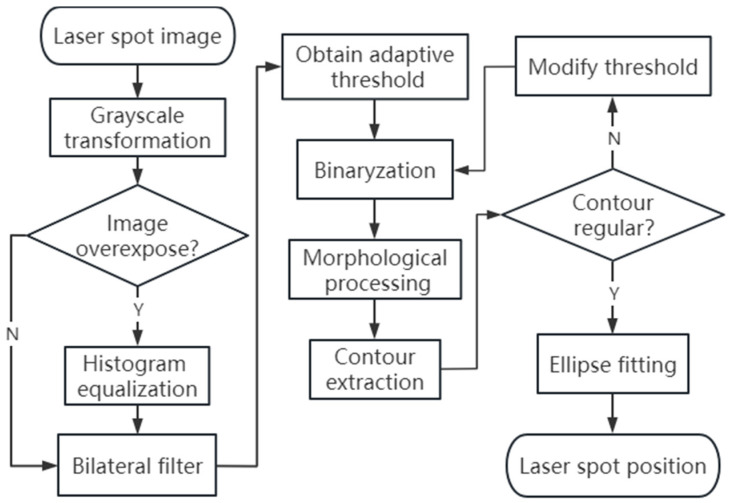
Process flowchart of the spot image.

The processing accuracy of the laser spot image will directly affect the measurement accuracy of orbital smoothness, and this paper designs a set of corresponding image processing processes for special annular spot images, as shown in Figure 8.

**Figure 9:**
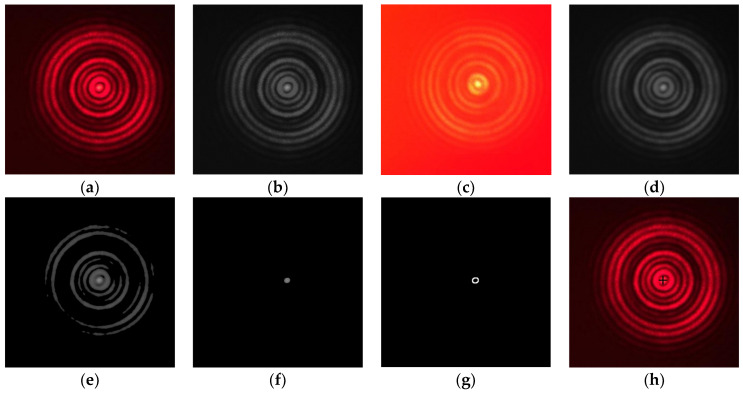
Laser spot image under different processing steps: (**a**) primitive light spots; (**b**) grayscale; (**c**) overexposure; (**d**) bilateral filtering; (**e**) threshold splitting; (**f**) split after the threshold adjustment is complete; (**g**) minimal bright spot contour; (**h**) the coordinates of the center of the spot are marked.

Due to the role of the single-wavelength narrowband filter in front of the detection target, the image obtained by the camera contains almost no interference with any background information. Figure 9a is a spot image in the field environment; in order to show the subsequent processing effect more clearly, the spot area image is magnified.

In order to improve the processing accuracy and operation speed of the program, the image acquired by the CMOS camera equipped with the detection target is a 3-channel color image with 640 × 480 resolution, and it is first grayscale processed into a single-channel image [21], as shown in Figure 9b. In addition, in the field measurement, it was found that, when the external ambient light is too strong, the camera image will be in an overexposed state, as shown in Figure 9c. Hence, the image is equalized to expand the difference of the image. Bilateral filtering is a filtering algorithm that comprehensively considers the difference in image spatial information and image pixel gray value, which plays a role in smoothing the high-frequency fluctuation signal while retaining the signal fluctuation with large value changes, which can realize the noise reduction processing of the image and retain the edge information of the image. The image after bilateral filtering processing is shown in Figure 9d. The image is segmented according to the difference in spot brightness, and the Otsu method is used to obtain the threshold and binarize the image [22], as shown in Figure 9e. Due to the influence of the transmission medium, the spot is uneven; therefore, there are many stray pixel blocks in the image after threshold segmentation, meaning the morphological treatment of removing spurious bright spots and then closing operation is adopted to repair the spot gap, as shown in Figure 9f. Contour extraction is the most critical step in the entire processing process and, in order to ensure that the outline information of the smallest bright spot in the center is found, this system seeks mainly to showcase the following: the total number of extracted contours, the number of pixels of a single outline, and the morphological characteristics of the contour of the three indicators for abnormal judgment. If there is no profile that meets the requirements, the binarization threshold must be modified and the subsequent steps must be repeated until the contour that meets the requirements is found. The binarized image after multiple threshold adjustments is shown in Figure 9g, and the outline extraction is obtained (Figure 9h). The contour of the smallest spot was elliptically fitted to find the coordinates of the center point after fitting, and the fitted coordinates are marked in Figure 9i as a black cross [23,24].

#### 3.3.2. Comparison of Spot Coordinate Extraction Methods

In traditional image processing, methods for extracting the center coordinates of light spots include the centroid method, edge detection, Hough transform, et cetera. In recent years, there have also been methods using emerging technologies, such as a method using neural networks proposed in reference [12].

In order to demonstrate the superiority of the proposed method in detecting circular light spots, a comparison was made between the proposed method and the other three commonly used methods. The experimental subjects were thirty circular spot images under different conditions. The average detection error of each method for the center coordinate of the annular spot was calculated and represented in Table 2. A result with a detection error greater than 10 pixels in the X or Y direction is considered a detection failure, and the detection failure rate of each method is represented in Table 3.

From the experimental results, it can be seen that the method proposed in this paper exhibits high measurement accuracy, but traditional detection methods have encountered significant detection errors or even detection failures in this experiment. This is due to the particularity of circular light spots, such as the energy distribution of ordinary light spots generally fitting with two-dimensional Gaussian functions. However, circular light spots are completely different. The method proposed in this paper has a relatively complex processing process, with a processing time of approximately 0.07–0.28 s for a single spot image, which is longer than the above processing methods. Therefore, it is not recommended to use this targeted method when processing ordinary spot images.

## 4. Performance Experiment of the Static Track Measurement System

**Figure 10:**
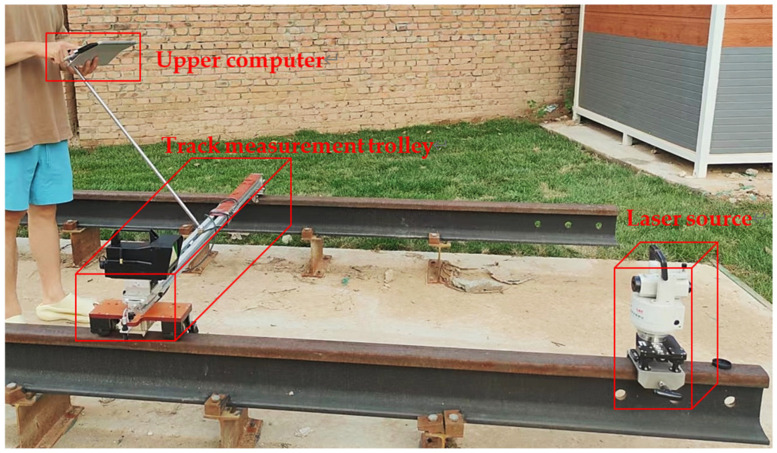
Experimental set-up. In this experiment, the repeatability accuracy of the proposed system is first measured to ensure that the laser source device is horizontal and aligned. Then, the automatic measurement mode is turned on in the upper computer software, and finally, the small car is pushed for mobile measurement. A total of 10 sets of track smoothness data were collected in a reciprocating manner, and the mean and standard deviation of each measurement point were calculated. Table 4 shows the time consumption of each part of the experiment.

This paper conducted on-site experiments to test the performance of the static track measurement system, and the experimental setup is shown in Figure 10. The core parameter configuration of various experimental instruments is as follows: the maximum power of the specially designed laser source device is 1000 mW, and the resolution of the displacement sensor is 1 μm. The resolution of the tilt sensor is 0.001°, the detection frequency of the photoelectric open light sensor is 25 Hz, the acquisition frequency of the CMOS camera is 30 Hz, the model of the embedded development board is LattePanda 3 Delta, and the upper computer uses the Windows 10 operating system.

The experimental object is a dedicated ballastless track approximately 100 m long (laid according to the Chinese national standard GB/T 38695-2020). After the experiment starts, the collimation operation is carried out. The laser source is installed at one end of the track and horizontally adjusted through a bubble device and leveling base. The track measuring car is then placed on the track, and the attitude is stable through the magnetic attraction structure and tilt sensor at the bottom. The horizontal position of the detection target head and the horizontal and pitch angles of the light source emission mechanism are adjusted to enable the light spot to vertically map to the center of the target surface of the detection target head. During the experiment, the embedded system installed on the track measuring car is responsible for collecting the values of all sensors, extracting coordinates, and converting units of the spot image. The upper computer is responsible for receiving the processed experimental data and completing tasks such as image display, saving, exporting, and anomaly analysis of the experimental data.

**Figure 11:**
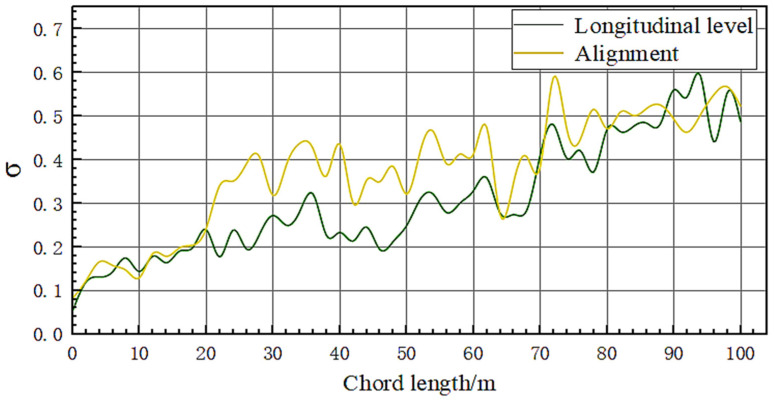
Standard deviation of alignment and longitudinal level.

Figure 11 shows the standard deviation data of the proposed orbit measurement system after multiple measurements at the same point. It can be seen that as the measurement distance increases, the variance of the measurement data shows an increasing trend, indicating a decrease in the repeatability accuracy of the orbit measurement system. Within 0–18 m, the repeatability accuracy of the proposed system for measuring track direction, and height can be maintained at a relatively low level. The repeatability accuracy of track measurement varies significantly between 20 and 100 m, and is significantly higher than that of high and low measurements.

**Figure 12:**
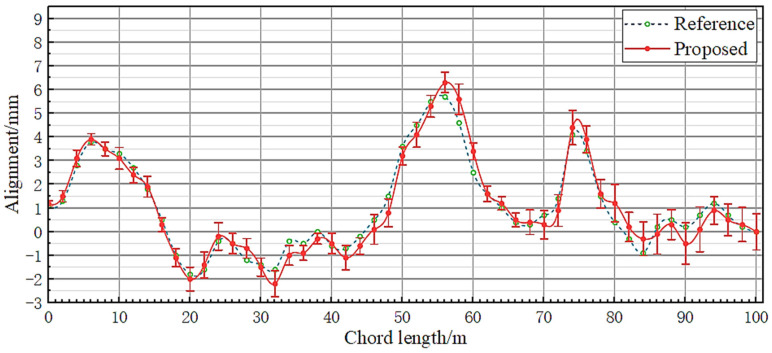
Alignment obtained from two types of track measurement systems.

**Figure 13:**
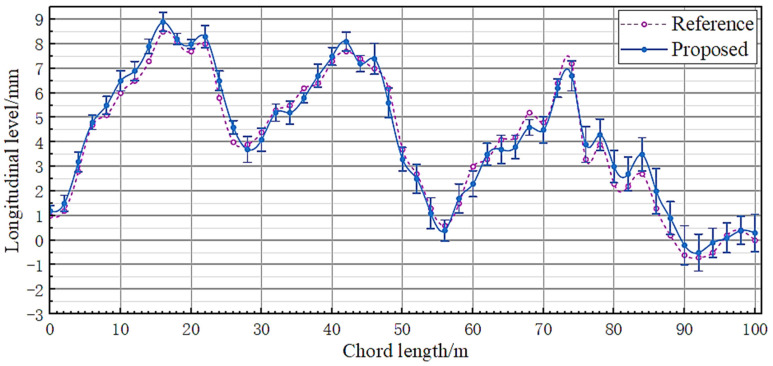
Longitudinal level obtained from two types of track measurement systems.

Figure 12 and Figure 13, respectively, show the measurement results of the proposed system and the comparison system on the alignment and longitudinal level, and represent the range of measurement points in the form of error bars on the measurement curve of the proposed system.

The comparative system used in this experiment is the Everbright’s GJY-T-EBJ-3 track detection system, which uses a fixed chord vector distance difference method for detecting alignment and longitudinal level. The detection accuracy is ±2 mm within a chord length of 300 m.

From the experimental data shown in Figure 10 and Figure 11, it can be seen that the measurement results of the two systems have a high degree of agreement. Within the 60 m chord length, the difference between the two data remains within 0.5 mm, and the maximum difference between the 100 m chord length does not exceed 1 mm. According to the current national standard GB/T 25021-2010 for track inspection vehicles in China, the proposed system meets the requirements of ±4 mm for alignment detection and ±3 mm for longitudinal level detection.

From the range data shown in Figure 12 and Figure 13, it can also be seen that, as the distance increases, the repeatability accuracy of single point measurement will decrease. In addition to the factors of the measurement system itself, it is also believed that the following points are important reasons for measurement errors:

(1) The experimental site is located near the city center and the impact of vibration effects caused by vehicle flow exists, but in most cases, there will not be a large number of vibration sources around the laying environment of actual tracks;
(2) The experimental time is during the day; therefore, even if the detection target made shading measures, it is still impossible to avoid the external ambient light entering the detection target. The changes in ambient light intensity during the measurement process can also have an impact on the final spot coordinates, resulting in deviations in the measurement results for the same point position;
(3) According to the experimental results in Section 3.1.2 of the paper, there is a limit to the compensation angle of the liquid wedge, and the absolute level of the outgoing beam cannot be guaranteed when the deviation angle of the laser source device is too large;
(4) The static laser measurement system does not have direct contact with the measurement point, and its measurement point is determined by two measuring pulleys. Therefore, there is no guarantee that the point locations measured using the two measurement methods will be exactly the same.

## 5. Conclusions

This paper focuses on the existing defects of the static detection system for track smoothness in laser datum. Three key technologies affecting the measurement accuracy and stability of the track measurement system are proposed and studied.

Automatic compensation technology of liquid optical wedge: In order to solve the problem of the horizontal deviation angle of the outgoing laser, this paper proposes the use of a liquid wedge as a compensator to compensate for the beam declination angle. A mathematical model of the refracted optical path of the laser beam on the inclined double liquid wedge is established, and rigorous mathematical derivation is carried out. Hence, the physical conditions for full compensation using the liquid double optical wedge are given.

Laser ring gate collimation technology: In order to solve the problem of the difficulty in collimation and focusing difficulty in the laser measurement system in long-distance measurement, a spatial phase optical modulation system is designed, and the divergence angle of the beam is expanded at the same time. Hence, the beam forms a set of light and dark ring spots by changing the phase information of the beam everywhere, which solves the problem that the optical system remote focusing time spot is easy to lose track of.

Spot image digitization technology: For the characteristic ring spot, a new image processing process for spot center positioning is designed, and the strategy of circular judgment is adopted to obtain the outline of the brightest spot in the center. Bilateral filtering, morphological processing, and other methods are also adopted to improve the image processing accuracy.

In this study, a comparative experiment is also designed, and the experimental results prove that the intelligent measurement system based on the above key technologies has high measurement accuracy and stability within a certain measurement distance. The intelligent rail measurement system proposed in this paper has exquisite structure, a simple principle, multi-sensor integration, and remote control with the host computer software, which can replace manpower measurement under certain conditions and improve the efficiency of the current railway track maintenance and overhaul. It is worth noting that there are still certain limitations to the usage of the track measurement system proposed in the manuscript. To ensure accuracy, it is required that the usage should be free of strong light, low temperature, and there should be no other significant vibration interference around. However, in general, track maintenance work is carried out during night when the train is stopped, and these limitations can be easily met.

## Figures and Tables

**Table 1:** The various physical quantities involved in the compensator’s internal optical path model and their representational significance.

$\alpha_{1}$	The incident angle of the laser beam on the upper surface of the first layer of liquid wedge
$\alpha_{1}^{\prime }$	The refraction angle of the laser beam on the upper surface of the first layer of liquid wedge
$\beta_{1}$	The incident angle of the laser beam on the lower surface of the first layer of liquid wedge
$\beta_{1}^{\prime }$	The refraction angle of the laser beam on the lower surface of the first layer of liquid wedge
$\alpha_{2}$	The incident angle of the laser beam on the upper surface of the second layer liquid wedge
$\alpha_{2}^{\prime }$	The refraction angle of the laser beam on the upper surface of the second layer liquid wedge
$\beta_{2}$	The incident angle of the laser beam on the lower surface of the second-layer liquid wedge
$\beta_{2}^{\prime }$	The refraction angle of the laser beam on the lower surface of the second layer liquid wedge
$\theta_{1}$	The angle between the laser beam and the measurement reference line after the first compensation
$\theta_{2}$	The angle between the laser beam and the measurement reference line after the second compensation
n	Refractive index of compensation liquid

**Table 2:** The detection results of circular light spots using different detection methods.

Error (Pixels)	Centroid	Hough Transform	Gaussian Fitting	Proposed
X offset	3.65	5.66	2.26	0.09
Y offset	2.71	4.58	2.74	0.04

**Table 3:** The failure rate of different detection methods for circular spot detection.

Centroid	Hough Transform	Gaussian Fitting	Proposed
16.6%	20%	13.3%	0%

**Table 4:** Time consumption of each part of the experiment.

TASK	Install Equipment	Collimation	Single Point Measurement	Single Group Measurement (100 m)	The Entire Experimental Process
Time Consuming	5 min	12 min	<1 s	5 min	1 h 20 min

## Data Availability

Not applicable.
